# Evaluation of the clinical effectiveness of bioactive glass (S53P4) in the treatment of non-unions of the tibia and femur: study protocol of a randomized controlled non-inferiority trial

**DOI:** 10.1186/s13063-018-2681-9

**Published:** 2018-05-30

**Authors:** Michael C. Tanner, Raban Heller, Fabian Westhauser, Matthias Miska, Thomas Ferbert, Christian Fischer, Simone Gantz, Gerhard Schmidmaier, Patrick Haubruck

**Affiliations:** HTRG – Heidelberg Trauma Research Group, Centre for Orthopaedics, Trauma Surgery and Spinal Cord Injury, Trauma and Reconstructive Surgery, Heidelberg University Hospital, Schlierbacher Landstrasse 200a, 69118 Heidelberg, Germany

**Keywords:** Non-union, Bioactive glass, Masquelet therapy, Bone regeneration, S53P4, Pseudarthrosis, Bone grafting

## Abstract

**Background:**

Treatment of non-union remains challenging and often necessitates augmentation of the resulting defect with an autologous bone graft (ABG). ABG is limited in quantity and its harvesting incurs an additional surgical intervention leaving the risk for associated complications and morbidities. Therefore, artificial bone graft substitutes that might replace autologous bone are needed. S53P4-type bioactive glass (BaG) is a promising material which might be used as bone graft substitute due to its osteostimulative, conductive and antimicrobial properties. In this study, we plan to examine the clinical effectiveness of BaG as a bone graft substitute in Masquelet therapy in comparison with present standard Masquelet therapy using an ABG with tricalciumphosphate to fill the bone defect.

**Methods/design:**

This randomized controlled, clinical non-inferiority trial will be carried out at the Department of Orthopedics and Traumatology at Heidelberg University. Patients who suffer from tibial or femoral non-unions with a segmental bone defect of 2–5 cm and who are receiving Masquelet treatment will be included in the study. The resulting bone defect will either be filled with autologous bone and tricalciumphosphate (control group, *N* = 25) or BaG (S53P4) (study group, *N* = 25). Subsequent to operative therapy, all patients will receive the same standardized follow-up procedures. The primary endpoint of the study is union achieved 1year after surgery.

**Discussion:**

The results from the current study will help evaluate the clinical effectiveness of this promising biomaterial in non-union therapy. In addition, this randomized trial will help to identify potential benefits and limitations regarding the use of BaG in Masquelet therapy. Data from the study will increase the knowledge about BaG as a bone graft substitute as well as identify patients possibly benefiting from Masquelet therapy using BaG and those who are more likely to fail, thereby improving the quality of non-union treatment.

**Trial registration:**

German Clinical Trials Register (DRKS), ID: DRKS00013882. Registered on 22 January 2018.

**Electronic supplementary material:**

The online version of this article (10.1186/s13063-018-2681-9) contains supplementary material, which is available to authorized users.

## Background

Fracture healing is a complex physiological process dependent on the intricate interaction of numerous partners [1]. Delayed or failed fracture healing can lead to enormous limitations in the quality of life due to pain, reduced mobility, and considerably longer duration of disease. In addition, non-union of a fracture can also lead to debilitating economic and social circumstances [2]. Despite recent research advances and modern treatment options, up to 30% of long bone fractures develop non-unions [1, 3, 4]. Especially the treatment of atrophic and infected non-unions, as well as large defect sizes, remains a challenge in trauma and orthopedic surgery. Treatment of segmental defects can be divided into two general categories: (1). bone transport and (2). bone filling [5, 6]. In defects larger than 4–6 cm in length the “gold standard” remains bone transport [6]; whereas, in segmental defects of between 2- to 5-cm bone filling has shown good clinical and radiological outcomes [5, 6]. Therefore, the induced membrane technique, also known as the Masquelet technique, was established [7–11]. It is a two-staged procedure; during the first step a vascularized membrane, containing growth factors and supporting the proliferation of human bone marrow stromal cells, is induced via a foreign-body reaction [7]. The second step involves surgically augmenting the membranous tube via an ABG, due to its osteostimulative and osteoconductive properties as well as its osteogenic potential [8, 10]. The most frequently accessed donor site remains the iliac crest. However, complications, such as donor-site morbidity, pain and quantitative limitations, are well-documented [12–15]. In recent years, the reamer/irrigator/aspirator (RIA) system has shown numerous advantages in harvesting autologous bone from the medullary canal of long bones [16]. Reaming debris became a reliable alternative as a source for autologous bone and the RIA system has shown decreased morbidity at the harvest site and none of the complications of the iliac crest site [17–21]. From a cellular aspect, mesenchymal stem cells harvested by the RIA system show significantly superior osteogenic differentiation and higher sensitivity towards stimulation with differentiation factors compared to mesenchymal stem cells isolated from iliac crest bone marrow [22, 23].

Evaluation of the outcome of non-union therapy remains challenging. A standardized approach was established by recent studies [11, 24] containing both radiological and clinical parameters. Clinical outcome can be determined via assessment of mechanical stability, pain associated with weight-bearing and the subjective health of patients [24]. Standard of care in the radiological assessment of bone healing and consolidation of non-union subsequent to treatment remains periodical conventional x-rays of the affected bone [11, 24–26]. Radiological union can be assumed when bridging of three out of four cortices is apparent. However, evaluation of osseous consolidation of non-unions relying merely on x-rays can be misleading. In a recent study by Akiho et al. [25] the authors compared conventional x-rays and computed tomography (CT) scans of pubic bone non-unions. Their data showed that CT scans were able to identify a larger number of delayed unions. Thus, even when osseous consolidation is presumed on x-ray, where there are numerous layers superimposed upon one another in both planes, persistent non-unions can only be detected reliably via CT scans [25]. However, utilization of CT scans is limited due to their higher radiation exposure. Therefore, a combination of both methods is beneficial to assess radiological outcome of non-union treatment. Promising new diagnostic modalities contributing to a timely identification of successful non-union treatment have been introduced in recent years. In particular, analysis of serum cytokine expression pattern was established as a valid method in the evaluation of the biological processes occurring during bone regeneration [27, 28]. In addition, dynamic contrast-enhanced magnetic resonance imaging (DCE-MRI) perfusion analysis after non-union treatment was able to successfully predict the outcome of non-union therapy [29]. Furthermore, the combination of DCE-MRI and contrast enhanced ultrasound (CEUS) was able to distinguish between infected and aseptic non-unions pre-operatively [30]. Hence, a combination of standardized and innovative diagnostic modalities contributes to a precise and timely identification of successful non-union treatment and, furthermore, helps identify patients at risk for infected non-unions.

Regardless of the source, harvesting of autologous bone necessitates an additional surgical intervention with a potential risk for associated complications and morbidities. Also, in some patients, either harvesting of autologous bone via RIA is anatomically impossible or they may have already have had both iliac crests depleted, or both may apply. Hence, alternative methods, such as allogenic bone, demineralized bone matrix and biomaterials designed as artificial bone graft substitutes, have been extensively studied, but found to be lacking in comparison to an ABG [31, 32]. In order to replace the ABG, the substituting biomaterial must be bioactive (the effect of the materials on cells that activate specific responses), degradable, osteoconductive and osteostimulative [33, 34]. BaG (S53P4) is such a material and is currently used as bone graft substitute and in the treatment of osteomyelitis [33]. BaG has osteostimulative properties; the release of calcium ions leads to formation of hydroxyapatite. It is also osteoconductive, serving as a scaffold for bone formation in vivo [33]. In addition, BaG has been shown to have antimicrobial properties due to its release of ions from its surface resulting in an increasing osmotic pressure and pH leading to a microenvironment unsuitable for microbial growth [35]. Therefore, S53P4-type BaG (53% SiO_2_, 4% P_2_O_5_, 23% Na_2_O and 20% CaO in wt%) is a promising material to employ as bone graft substitute in context with non-union therapy.

The current study is a randomized controlled trial (RCT) regarding the non-inferiority of the effectiveness and safety of the use of BaG (S53P4) as bone graft substitute in Masquelet therapy for treating large-sized-defect non-unions of the tibia and femur in comparison to the standard therapy. The study protocol for the RCT is described in the present manuscript.

## Methods/design

### Objectives

The primary objective of this study is to evaluate the non-inferiority of the clinical effectiveness of BaG as a bone graft substitute in Masquelet therapy when compared to present standard Masquelet therapy using an ABG in combination with ceramic bone substitutes, such as tricalciumphosphates, to fill the bone defect. Secondary objectives include subjective patient quality of life directly post-operative as well as during the time of recovery, documentation of perfusion of the bone graft using CEUS as well as DCE-MRI [26, 29]. Furthermore, patient data (such as smoking status, drug abuse, profession, time necessary to return to work and pre-existing condition) will be assessed and evaluated. Therefore, the results from the current study will facilitate the evaluation of BaG as a bone graft substitute regarding objective parameters associated with consolidation as well as subjective parameters associated with the patients’ quality of life. Furthermore, patients who are at risk for unsuccessful treatment with one or the other approach might be identified. Hence, results from the study will contribute to thoroughly assess whether BaG is a suitable bone graft substitute in non-union therapy using the Masquelet method.

### Study design

This is a registered, prospective, single-center, two-arm, parallel-group, randomized controlled non-inferiority trial (DRKS00013882).

### Inclusion and exclusion criteria

Patients older than 18 years who suffer from tibial or femoral non-unions with a segmental bone defect of 2–5 cm and who are receiving Masquelet treatment will be included into the study after giving informed consent. Patients who do not agree to participate in the study, who are not applicable for harvesting autologous bone using the RIA system, who are not able to give informed consent and patients receiving an amputation because of persistent infection or extended soft tissue defects will be excluded from the study.

### Setting

The study is carried out at the Department of Orthopedics and Traumatology at Heidelberg University (level 1 trauma center). Surgical treatment of non-union is established and a standardized follow-up setting is development to monitor response to the treatment and clinical consolidation [4, 11, 24].

### Randomization

Due to the sample size a block randomization procedure with randomly chosen block sizes is used to assign participants to each group (1:1 ratio), resulting in one intervention and one control group. This method helps in maintaining the balance of treatment assignment while reducing the potential for selection bias [36]. Randomization is performed by an employee not involved in treatment, assessment or data collection regarding the present study using opaque, sealed envelopes.

### Surgical treatment

**Fig. 1:**
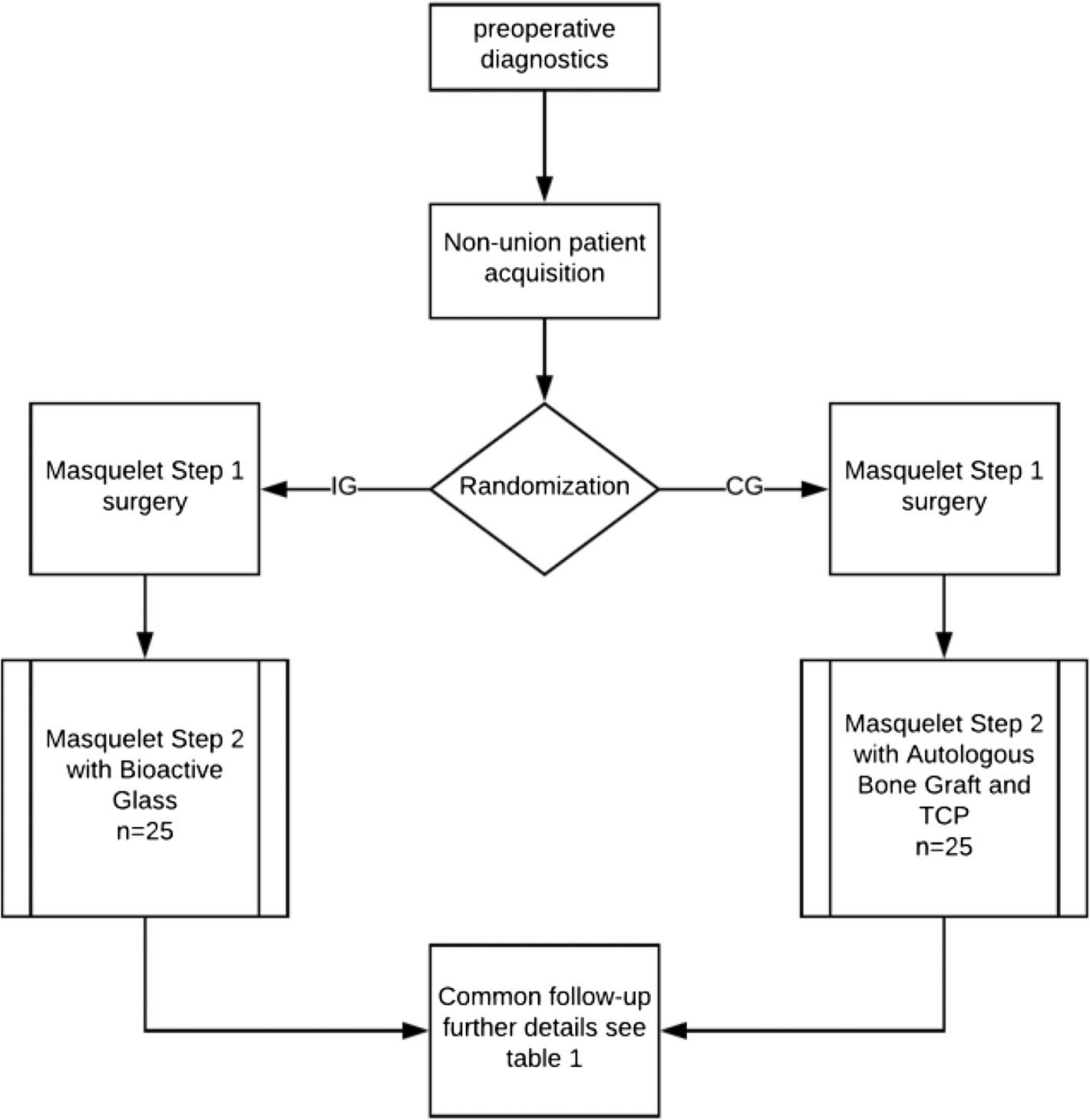
Flow chart of the Masquelet therapy and treatment pattern of included patients

After information and randomization, all patients receive contrast-enhanced ultrasound sonography (CEUS) pre-operatively to evaluate local perfusion. Hereafter, they are scheduled for Masquelet therapy of the non-union. Masquelet therapy is based on the principles of the “diamond concept” [37] and is a two-step procedure (step I and step II). During the first step, radical debridement of the non-union, non-viable bone and surrounding tissue is performed [38]. The resulting segmental bone defect is subsequently filled with polymethylmethacrylate (PMMA), which induces a foreign-body reaction, resulting in a vascularized Masquelet membrane [7]. In addition, multiple bone and soft tissue samples are harvested for microbiological examination. The first step is repeated until all samples are sterile. Once sterile, the spacer is left in situ for 6 weeks to guarantee a fully grown Masquelet membrane [7, 10]. In a second step the spacer is removed while leaving the membrane unimpaired and the resulting bone defect filled with either autologous bone and tricalcium phosphate (control group) or BaG (S53P4) (study group). Due to the decreased morbidity at the harvest site and significantly superior osteogenic differentiation and higher sensitivity towards stimulation with differentiation factors harvesting of autologous bone graft will be performed with the RIA system [39]. However, if the quantity of the reaming material threatens to be insufficient, additional harvesting of the iliac crest might be necessary to achieve sufficient filling of the osseous defect. De novo osteosynthesis utilizing plates, nails or external fixators is performed depending on the biomechanical stability during the first or second step of the Masquelet therapy. The eligibility of the utilized method of osteosynthesis will be based on anatomical premises as well as morphology of the non-union and will be carefully evaluated pre-operatively. A flow chart of the surgical treatment is depicted in Fig. 1. Post-operatively, all patients regardless of the method of fixation will be treated with partial weight-bearing of 20 kg for 6 weeks; afterwards patients will gradually increase weight-bearing with approximately 10 kg per week until full weight-bearing is achieved.

### Follow-up

Subsequent to the operative therapy, all patients will receive the same follow-up procedures. Follow-up at our institution is standardized and all procedures and diagnostics are based solely on medical indications. Initial radiological and clinical evaluation of the surgical treatment will be performed on day 2 after surgery. Discharge from the hospital will be realized as soon as soft tissue conditions, patient mobility and pain level allow it. Afterwards, patients will receive physiotherapy on a regular basis of at least twice a week. Clinical and radiological follow-up visits at our hospital are planned at 6 weeks, 3, 6, 9, 12 and 24 months post-operatively, following the standardized procedure for non-union patients treated in our hospital (see Table 1) [27]. By using a questionnaire pre-operatively and post-operatively after 3, 12 and 24 months, patients can give information on pain, mobility of limbs and life quality (SF-12) during the course of treatment. General patient data, such as profession, Body Mass Index (BMI), risk factors, medication, pre-existing conditions, previous surgeries and accident data will be obtained pre-operatively. Subsequent to step II, the outcome of therapy will be evaluated based on clinical and radiological examination. Potential for vascularization will be evaluated pre-operatively and at 12 weeks subsequent to step II via CEUS using the established protocol [26]. After 12 weeks, patients also receive DCE-MRI to assess vitality of the graft as a previous study has shown that DCE-MRI perfusion analysis after non-union surgery predicts successful outcome [29].

**Table 1:** Structure of clinical and radiological follow-up

	Pre-op	2 D post-op	6 W post-op	3 M post-op	6 M post-op	9 M post-op	12 M post-op	24 M post-op
Clinical examination	x	x	x	x	x	x	x	x
X-ray	x	x	x	x	x	x	x	x
DCE-MRI				x				
CT							x	
CEUS	x			x				
Questionnaire (SF-12)	x			x			x	x
Laboratory work	x	x	x	x	x	x	x	x

*Abbreviations*: *pre-op* pre-operative, *post-op* post-operative, *D* days, *W* weeks, *M* months, *DCE-MRI* dynamic contrast-enhanced MRI, *CT* computer tomography, *CEUS* contrast-enhanced ultrasound, *SF-12* 12-item Short Form health survey

After 12 months, a CT of the affected bone will be performed to further evaluate osseous consolidation. Patients in both study groups will be declared responder/non-responder due to radiological signs of consolidation and clinical signs of mechanical stability and full weight-bearing. Furthermore, blood samples will be obtained during the course of treatment and analyzed regarding parameters of infection, growth factors and cytokines associated with angiogenesis. Previous studies have shown that analysis of serological cytokine expression pattern is a valid tool in evaluation of the potential for angiogenesis and effectiveness for additional non-union therapy [27, 28]. After 12 and 24 months, results of the groups will be statistically analyzed and compared. Duration of patient enrollment will be 2 years. Data will be stored and monitored using pseudonyms. Only PH and MCT have access to the full names of the participants. Follow-up will be 2 years with data analysis after 1 year and 2 years. The duration of the study is 4 years (Table 1).

### Primary outcome measure

The primary endpoint of the study is union achieved 1 year after surgery by evaluation of x-rays in two planes (defined as cortical bridging of at least three out of four cortices) and CT scan [40]. The radiographic datasets will be blinded and evaluated by a group of experienced orthopedic surgeons.

### Secondary outcome measure

Secondary endpoints include subjective evaluation of the quality of life (assessed by the 12-item Short Form health survey (SF-12) questionnaire) and pain (Visual Analog Scale (VAS) of affected patients. In addition, perfusion of the graft is evaluated using CEUS and DCE-MRI and compared between groups. Expression patterns of inflammatory and angiogenic cytokines are evaluated during the course of the study and compared between groups regarding possible differences. Additionally, union achieved 2 years after surgery will be evaluated based on x-rays in two planes (defined as cortical bridging of at least three out of four cortices) and possible differences regarding socioeconomic factors (time necessary to return to work, time of recovery) are assessed and compared between groups.

### Criteria that lead to termination of study

Data of included patient will be continuously monitored regarding outcome and unexpected risk for participating patients. If initial data indicates either an inferior outcome of patients included into the study group, or an increased risk for patients of the study group that is potentially harmful for patients, the study will be terminated. Furthermore, if patients want to withdraw their consent to the study, they will be excluded from the current study. Withdrawal from the study will not impact the quality of the medical treatment of patients.

### Statistical analysis

Statistical calculation will be conducted with R version 3.4.3 [41], figures will be created using the package “ggplot2” [42]. Receiver operator characteristics analysis will be performed via the “pROC” package [43]. Correlation analyses will be performed between all variables. Non-parametric tests (Mann-Whitney *U* test for independent variables, Wilcoxon signed-rank test for dependent variables) will be utilized to investigate location shifts between groups. Differences between categorical variables will be examined via the chi-square test. The Kruskal-Wallis test will be used to assess differences in more than two independent samples. To evaluate the predictive power of variables regarding the criterion “consolidation” adjusting for potentially clinically relevant covariates logistic regression models will be set up and constructed via backwards selection. Analogous to our previous studies [44–46], predictive performance will be assessed through estimation of the models AUC (area under the curve) of the corresponding ROC curve and AIC (Akaike information criterion). Continuous variables will be expressed as absolute mean concentrations ± SD (standard deviation) and the level of significance (α) is set at 5%.

### Sample size determination

Currently, there are no comparable studies available in the academic literature. In order to determine the necessary sample size data from a previous study was utilized (Author: Haubruck P, Tanner M, Vlachopoulos W, Hagelskamp S, Miska M, Ober J, Fischer C, Schmidmaier G. Title: Comparison of the clinical effectiveness of Bone Morphogenic Protein (BMP) -2 and -7 in the adjunct treatment of lower limb non-unions: a matched pair analysis. Submitted 2018). In this study, a similar patient collective suffering from non-unions of the same anatomical region being treated with Masquelet therapy were evaluated regarding their osseous consolidation. Based on our previous study we performed the sample size calculation for the binary-outcome non-inferiority trial in R [41] using the package “‘SampleSize4ClinicalTrials” by Hongchao Qi. Additionally, assuming an alpha level of .05 and a power of .90 as well as an equal number of subjects in the experimental and control groups we estimated that 50 patients in total (25 patients each group) to be required. Intervention group sizes will match this determined sample size.

## Discussion

This study aims to investigate into the non-inferiority of the clinical effectiveness of BaG (S53P4) as a bone graft substitute in Masquelet therapy compared to the standard Masquelet therapy using autograft.

BaG has been established in previous case series and in vivo animal studies, but not yet in RCTs as a promising biomaterial due to both its osteostimulative, osteoconductive (serving as a scaffold for bone formation in vivo) and antimicrobial properties [33]. In particular, after implantation a surface reaction occurs, resulting in formation of a calcium phosphate layer [33, 47]. Release of various ions increases local pH and osmotic pressure, then a silica gel layer is formed on the surface of the biomaterial and amorphous calcium phosphate precipitates on this layer [33]. Thereafter, crystallization to natural hydroxyapatite occurs, which starts the activation of osteoblasts and initiates the formation of new bone [33, 48]. During this process, new bone is constituted, the BaG absorbed and the antibacterial microenvironment maintained due to a persistently increased pH [33]. Hence, BaG as an artificial bone substitute might contribute to successful non-union treatment by both osteostimulative bone regeneration and prevention of an infection via its antimicrobial properties. Furthermore, implantation of BaG prevents the surgical intervention necessary for harvesting of the ABG and, therefore, might contribute to a lower complication rate and lower comorbidities associated with Masquelet therapy. A potential limitation of the planned study lies in the utilization of different methods of osteosynthesis that might influence the outcome of non-union treatment. However, a study by Vallier et al. compared the results between plate fixation and intramedullary nail fixation of tibial shaft fractures. The authors concluded that rates of union, infection and secondary procedures were similar [49]. In addition, all included patients will employ the same post-operative weight-bearing pattern. Therefore, we believe that the influence of different methods of osteosynthesis on the findings of the planned study to be minimal. The results from the current study will help evaluate the clinical effectiveness of this promising biomaterial in non-union therapy. Our hypothesis is that S53P4-type BaG will have the same rate of consolidation as autologous bone when used in the second step of the Masquelet therapy. Furthermore, we assume that the rate of perioperative infection in patients treated with S53P4-BaG will be reduced compared to the control group and that patients of the study group will have fewer post-operative complications and morbidities. The results of the study should, therefore, help investigate the potential benefits and limitations regarding the use of S53P4-BaG in Masquelet therapy. Data from the study will increase the knowledge about S53P4-BaG as a bone graft substitute as well as identify patients who might benefit from Masquelet therapy using this type of BaG and those who are more likely to fail. Ultimately, the current study might contribute to an improvement in the quality of non-union treatment.

### Trial status

The RCT recruitment and surgical treatment are planned from April 2018 until April 2020. Follow-up will be conducted over 24 months for each included patient. Data analysis and evaluation will be performed after 12 months and 24 months. The study will be halted if the study group shows severe disadvantages after 12 months. Final results of this study will be published.

## Additional file

Additional file 1: Standard Protocol Items: Recommendations for Interventional Trials (SPIRIT) 2013 Checklist: recommended items to address in a clinical trial protocol and related documents. (DOC 119 kb)

## Abbreviations

ABG: Autologous bone graft
BaG: Bioactive glass
CEUS: Contrast enhanced ultrasound
CT: Computed tomography
DCE: Dynamic contrast-enhanced
MRI: Magnetic resonance imaging
MSC: Mesenchymal stem cells
PMMA: Polymethylmethacrylate
RCT: Randomized controlled trial
RIA: Reamer/irrigator/aspirator
